# Calnexin, More Than Just a Molecular Chaperone

**DOI:** 10.3390/cells12030403

**Published:** 2023-01-24

**Authors:** Tautvydas Paskevicius, Rabih Abou Farraj, Marek Michalak, Luis B. Agellon

**Affiliations:** 1 Department of Biochemistry, University of Alberta, Edmonton, AB T6G 2R3, Canada; 2 School of Human Nutrition, McGill University, Sainte Anne de Bellevue, QC H9X 3V9, Canada

**Keywords:** calcium binding protein, cell signaling, endoplasmic reticulum, molecular chaperone, protein–protein interactions

## Abstract

Calnexin is a type I integral endoplasmic reticulum (ER) membrane protein with an N-terminal domain that resides in the lumen of the ER and a C-terminal domain that extends into the cytosol. Calnexin is commonly referred to as a molecular chaperone involved in the folding and quality control of membrane-associated and secreted proteins, a function that is attributed to its ER- localized domain with a structure that bears a strong resemblance to another luminal ER chaperone and Ca^2+^-binding protein known as calreticulin. Studies have discovered that the cytosolic C-terminal domain of calnexin undergoes distinct post-translational modifications and interacts with a variety of proteins. Here, we discuss recent findings and hypothesize that the post-translational modifications of the calnexin C-terminal domain and its interaction with specific cytosolic proteins play a role in coordinating ER functions with events taking place in the cytosol and other cellular compartments.

## 1. Introduction

The endoplasmic reticulum (ER) is organized as a continuous membrane network of branching tubules and flattened sacs that envelop a single lumen. The ER performs a plethora of functions in cells, including lipid and steroid synthesis, Ca^2+^ storage and signaling, protein synthesis and maturation involving protein folding and post-translational modification [1,2,3]. Human cells express approximately 10,000 different proteins at any given moment [4]. More than a third of all of these proteins are synthesized on ER membrane-bound ribosomes where proteins are either destined for residence in the ER, plasma membrane, Golgi apparatus, lysosomes or secreted from the cell [5]. Even though the native structure and conformation of a given protein is largely determined by its amino acid sequence [6], many newly synthesized proteins require assistance by molecular chaperones to reach their native fold at a biologically relevant time scale [7]. To help facilitate proper folding and quality control, the ER employs two major folding systems: the general pathway that is mediated by BiP (the ER homolog of the 70-kDa heat shock protein, Hsp70) together with protein disulfide isomerase PDIA1, and the N-linked glycoprotein pathway which is governed by lectin chaperones calnexin and calreticulin, commonly referred to as the calnexin/calreticulin cycle [8]. Both calreticulin and calnexin share structural similarity with respect to their lectin-like domains with calreticulin being an ER lumen-resident protein while calnexin is a type I integral ER membrane protein with a transmembrane helix and cytosol-exposed C-terminal domain. This review is focused on the role of calnexin not only as an ER chaperone involved in the protein quality control pathway but also on the emerging view on its importance in coordinating ER and cytosolic events via the unique interactions of calnexin with a variety of proteins at the ER–cytosol interface [9,10,11,12,13,14].

## 2. Discovery of Calnexin

In 1982 a novel ER-associated protein with an apparent mass of 90 kDa was detected using polyclonal antiserum raised against membrane fractions of rough ER [15]. Cell culture immunofluorescence analysis using these anti-rough ER antibodies revealed an extensive, reticular network of membrane occupying the entire cytoplasm and extending to the nuclear membrane [15]. However, it was not until a decade later when the 90 kDa phosphoprotein (referred to as pp90) was identified as being associated with ER signal sequence receptor complexes in canine pancreatic microsomes [16]. Two other research groups simultaneously reported the identification of p88 (reported as an 88 kDa protein) in transient association with partially assembled class I major histocompatibility molecules in murine lymphoma cell lines [17] and IP90 (reported as a 90 kDa intracellular protein) interacting with T-cell antigen and B-cell antigen receptor complexes [18]. Molecular cloning and characterization of the canine IP90 cDNA revealed that its encoded protein was a type I ER membrane protein [16]. Due to its Ca^2+^-binding properties [16] and a high degree of amino acid sequence similarity with calreticulin [19], a major Ca^2+^-binding ER resident protein, the pp90 protein was named calnexin [16,20,21,22]. Since then, calnexin has been observed to interact transiently with a wide array of nascent membrane or soluble N-linked glycoproteins.

**Figure 1:**
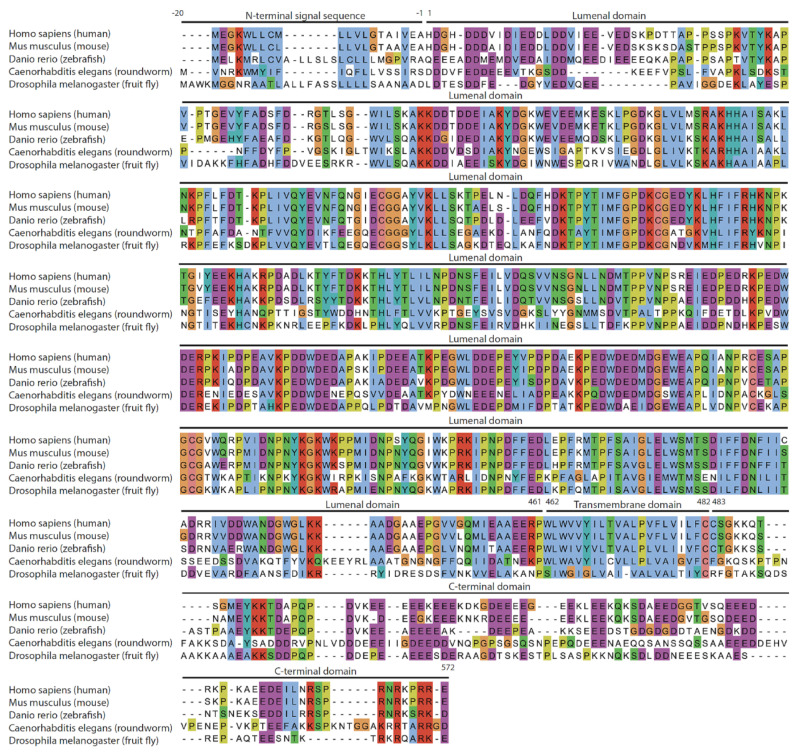
Amino acid sequence alignment of calnexin from different species. The default Clustal X color scheme was used for similar/identical amino acid residues [42]: blue for hydrophobic, red for positively charged, magenta for negatively charged, green for polar, cyan for aromatic, pink for cysteine, orange for glycine, yellow for proline and no color for amino acid residues that are not conserved. Dashes represent gaps in the amino acid sequence. The N-terminal signal sequence, intraluminal domain, transmembrane domain and C-terminal domain are indicated based on human calnexin protein topology. The numbering of amino acid residues referred to in the figure as well as the text corresponds to the mature human calnexin protein. Alignment was performed using Multiple Alignment using Fast Fourier Transform (MAFFT) high speed multiple sequence alignment program (https://toolkit.tuebingen.mpg.de/tools/mafft; accessed 31 August 2022). Jalview [43] was used to visualize amino acid alignment. Additional calnexin sequences from different species are shown in Supplementary Figure S1.

Calnexin, together with calreticulin and ERp57 (also known as PDIA3) [23], forms the core components of a pathway that facilitates the folding and quality control of newly synthesized proteins with N-linked carbohydrate side chains [24,25]. This folding pathway has been widely studied and therefore well characterized and described in several excellent review articles [26,27,28,29,30,31]. Today it is well established that calnexin is ubiquitously expressed in all cells containing the ER membrane. It is highly conserved among different species (Figure 1), with its intraluminal domain responsible for chaperone function displaying the highest level of conservation and indicating the evolutionary importance of the chaperone domain of calnexin. Calnexin can be found distributed within different ER membrane subdomains, including a variety of ER membrane contact sites [32] such as perinuclear rough ER contacts with the ribosome–translocon complex [33,34], smooth ER, nuclear envelope and the mitochondria/ER contact sites (also referred to as the mitochondria-associated membrane [12,35]. The characteristic distribution of calnexin molecules within ER membranes is controlled by the post-translational modifications including palmitoylation and phosphorylation at its C-terminal domain, which are discussed later. Moreover, it was initially reported that calnexin is present on the cell surface of immature thymocytes in a complex with the CD3 antigen due to incomplete ER retention [36,37]. Other studies have detected small amounts of calnexin on the cell surface of various cell types [38]; the redistribution of calnexin between ER and plasma membranes was proposed to be controlled by the state of calnexin C-terminal domain phosphorylation and association with phosphofurin acidic cluster sorting protein 2 (PACS-2) [39]. Moreover, plasma membrane localization of calnexin has been detected in cancerous tumors such as oral squamous cell carcinoma and melanoma [40] while another study reported calnexin as being secreted in the serum of lung cancer patients, making calnexin a possible sero-diagnostic marker [41].

### 2.1. Calnexin-Deficient Animal Models

Much of the early work on calnexin focused on biochemical and cellular aspects. Thus, animal models lacking calnexin were created to gain insight into its physiological importance. *D. melanogaster* has three genes encoding calnexin among which calnexin99A has the highest similarity with mammalian calnexin [44]. Mutations in calnexin99A affected the maturation and function of rhodopsin, which in turn led to age-dependent retinal degeneration [44]. Additionally, calnexin99A mutants displayed impaired Ca^2+^ buffering, which contributed to the development of the retinal degeneration possibly due to Ca^2+^ toxicity [44]. Inactivation of the calnexin gene in *C. elegans* resulted in developmental and reproductive defects that were temperature sensitive [45]. These mutant worms also exhibited growth impairment under calcium insufficiency [45]. Furthermore, RNAi-mediated silencing of the calnexin gene resulted in suppressed necrotic-like cell death [46]. In *D. rerio* (zebrafish), calnexin is required for the development of the mechanosensory system called the lateral line [47]. Upon the knockdown of calnexin, zebrafish exhibited reduced posterior lateral line cell proliferation and increased ER stress-dependent apoptosis [47].

Two independent calnexin-deficient mouse strains have been generated [48,49]. Unlike calreticulin deficiency which is embryonic lethal due to impaired Ca^2+^-dependent transcriptional regulation resulting in defective cardiac development [50], calnexin-deficient mice were born live but exhibited a high degree of neonatal lethality [48,49]. Calnexin-deficient mice were born with neurological disorders that included severe ataxia [48,49]; however, one functional calnexin allele is sufficient to prevent this defect [48]. The first study described the high early postnatal mortality of calnexin-deficient mice [49] and surviving mice exhibited ataxia due to the substantial loss of motor nerve fibers. The subsequent study by Kraus et al. [48] found however that surviving calnexin-deficient mice were fertile, had a normal life span, but were 30–50% smaller than their wild-type littermates. These mice developed peripheral neuropathy abnormalities manifesting as gait disturbance with instability, splaying of the hind limbs, tremors and a rolling walk but no reduction in the numbers of neuronal fibers was apparent [48]. Additionally, another mouse strain expressing a mutant form of calnexin-lacking amino acid residues 103–242 (encoded by exons 4–6), which deleted the regions involved in disulfide bond formation (Cys^141^ and Cys^175^) and carbohydrate binding (Tyr^145^ and Lys^147^), exhibited features that were identical to the calnexin-deficient mice, suggesting that the loss of chaperone function was responsible for the observed neurological defect [48,49]. Consistent with this idea, mice that only express the truncated version of calnexin lacking the C-terminal domain do not have apparent disturbances in motor function and display normal motor and sensory nerve conduction velocities [51]. Surprisingly, calnexin-deficient mice display no apparent aberrations in immune system development and function [48].

It is interesting to note that despite the ubiquitous presence of calnexin in all cells that possess an ER network, the loss of calnexin in the whole organism does not produce a common phenotype but rather manifests in a variety of phenotypes.

### 2.2. Calnexin as a Molecular Chaperone

It is predicted that more than 30% of all eukaryotic proteins are glycoproteins with more than 90% of these containing N-linked sugars [52]. The folding and maturation of newly synthesized glycoproteins in the ER is assisted by calnexin and its soluble ER lumen-resident homolog, calreticulin. Whereas calnexin binds to glycans in protein domains that are close to membranes, calreticulin interacts with glycans that extend deeper into the ER lumen [31]. Immediately after the nascent polypeptide exits the translocon and enters the ER lumen, oligosaccharyltransferase transfers dolichol-pyrophosphate-bound branched core glycan to the sidechain nitrogen of the asparagine residue of the N-glycosylation consensus sequence motif (Asn-X-Ser/Thr, where X is any amino acid except for proline) [53]. The branched core oligosaccharide is comprised of three terminal glucoses, nine mannoses, and two N-acetyl-glucosamines (Glc_3_Man_9_GlcNAc_2_). After the glycosylation, N-linked glycans are then processed by the subsequent action of endoplasmic reticulum (ER) glucosidases I (GI) and II (GII) removing the outer and middle glucose residues, respectively. As a result, the processing intermediate containing the single terminal glucose moiety is specifically recognized by calnexin and calreticulin [24,54,55]. Additionally, using their extended arm-like P-domains, calnexin and calreticulin recruit ERp57, cyclophilin B and ERp29 (also known as PDIA9) [56,57,58,59,60] to promote glycoprotein folding and maturation. Subsequently, the remaining innermost glucose moiety is removed by GII releasing the glycoprotein substrate from the calnexin–calreticulin complex. If the protein is correctly folded at this point, it is released from the ER and transferred to the Golgi apparatus to continue its journey along the secretory pathway. However, if the protein has not reached its native three dimensional conformation, it is re-glucosylated by the ER-folding sensor uridine diphosphate (UDP)-glucose:glycoprotein glucosyl transferase (UGGT) facilitating the re-association with calnexin and calreticulin for an additional round of the folding cycle [61]. Thus, the protein can re-enter the calnexin/calreticulin cycle multiple times until the native conformation is reached.

If numerous folding cycles fail to properly fold the protein, the misfolded protein is marked for degradation. Terminally misfolded glycoproteins and unassembled oligomers are retro-translocated to the cytosol and are degraded by the ubiquitin-proteasome system, a process known as ER-associated degradation (ERAD) [62]. This process is regulated by ER α-mannosidase I [63] and ER degradation-enhancing α-mannosidase-like protein (EDEM) [64]. Proteins undergoing multiple calnexin/calreticulin cycles are eventually subjected to mannose trimming by ER α-mannosidase I converting Man_9_GlcNAc_2_-glycans to Man_8_GlcNAc_2_ [65]. The slow kinetics of this enzyme essentially act as a timer for repeated glycoprotein folding cycles [66]. As a result, Man_8_GlcNAc_2_ becomes less efficient UGGT substrate [27] and instead is recognized by EDEM which acts as a signal triggering ERAD [67]. EDEM directly interacts with calnexin and accepts terminally misfolded glycoproteins upon mannose trimming [68,69]. This is followed by retro-translocation into the cytosol where the misfolded proteins are polyubiquitinated which are then degraded by the cytosolic 26S proteasome [70].

In the event of persistent ER stress and the extensive accumulation and aggregation of misfolded proteins, a selective form of autophagy named ER-phagy is used to ensure the timely removal of damaged ER [71]. During this process excessive or damaged portions of ER are fragmented and sequestered through ER-phagy receptors by double-membrane autophagic vesicles that eventually fuse with lysosomes for degradation [71]. It has been shown that calnexin makes a stable complex with the ER-phagy receptor FAM134B [72]. In collagen-producing cells calnexin acts as a co-receptor recognizing misfolded procollagen molecules in the ER lumen triggering FAM134B to recruit and bind the autophagosome membrane-associated protein LC3. In turn, this ER-phagy complex delivers a targeted portion of ER that contains both misfolded procollagen and calnexin to the lysosome for degradation [72]. Additionally, it has been shown that calnexin-FAM134B can facilitate the clearance of proteasome-resistant polymers of α1-antitrypsin Z in ER through a different vesicular transport pathway [73]. This pathway is known as the ER-to-lysosome-associated degradation (ERLAD), since ER-derived vesicles containing misfolded proteins are not encapsulated by autophagosomes but instead fuse with endosomes for degradation [73].

It was initially proposed that calnexin was an important factor in the development of the immune system, considering its importance in the folding and quality control of secreted and membrane-bound glycoproteins. It has been suggested that calnexin is involved in the folding and assembly of major histocompatibility complex (MHC) class I, although one study using a cultured cell model showed that these proteins can fold properly in the absence of calnexin [74,75,76,77,78,79,80]. Additionally, calnexin participates in MHC class II [81], T-cell antigen receptor (TCR) [21,82,83,84,85] and B-cell antigen receptor (BCR) [86,87,88] assembly and maturation. However, since mice with whole-body calnexin deficiency display normal immune function, it is apparent that calnexin is not essential for the development of the immune system in this species [48].

## 3. Structure of Calnexin

**Figure 2:**
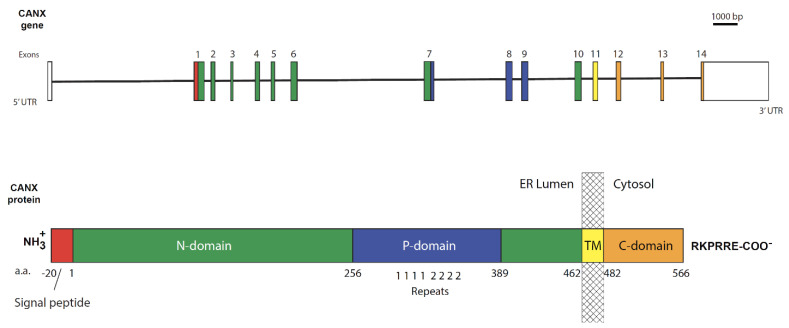
Schematic linear sequence of the calnexin gene and its encoded protein. The human calnexin gene is located on a forward strand of distal end of the long arm of chromosome 5 and is comprised of 15 exons (of which 14 are protein-coding exons) transcribed to a mature transcript of 4915 bp that is translated into a 592 amino acid residue polypeptide. Linear schematic representation of the calnexin protein and the corresponding exons encoding the specific protein domains of calnexin. The white boxes in the gene schematic diagram correspond to the untranslated regions of the first and last exons. The calnexin protein schematic diagram shows the signal peptide (red), the luminal domain (green for the N-domain and blue for the P-domain), the transmembrane domain (TM, yellow) and the cytosolic C-terminal domain (orange). The hatched box represents a portion of the ER membrane. Four repeats of Motif 1 and four repeats of Motif 2 are labelled as “11112222” and depict the proline-rich amino acid sequence repeats in the P-domain. The ER retention signal (RKPRRE) is shown as the most distal C-terminal amino acid sequence.

The gene for human calnexin (*CANX*) is located towards the distal end of the long arm of chromosome 5 (5q35.3 locus) spanning ~33 kbp and comprised of 15 exons [89] (Figure 2). It makes a mature transcript of 4915 bp that is translated into a 592 amino acid residue polypeptide.

**Figure 3:**
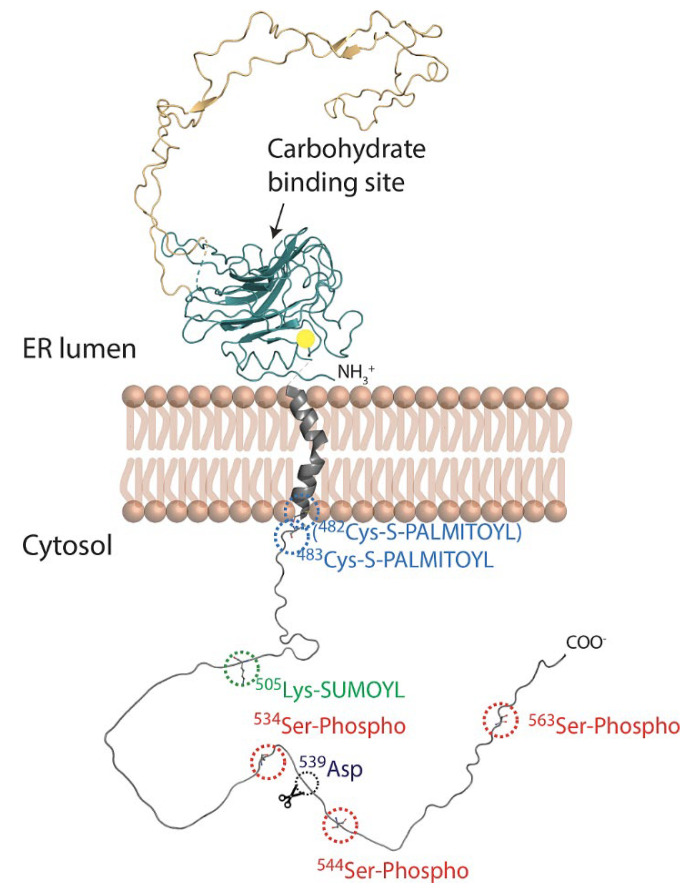
Schematic representation of full-length membrane-embedded calnexin. Calnexin (Protein Data Bank DOI: 10.2210/pdb1JHN/pdb) has three domains: luminal domain (comprised of N and P subdomains), transmembrane domain and C-terminal domain (no experimental structural information available). The yellow circle depicts a bound Ca^2+^ ion. The numbering of amino acid residues in the C-terminal domain is relative to the mature N-terminus. The transmembrane domain was modelled based on molecular dynamics simulation performed by Lakkaraju et al. [34] and shows that Pro^494^ introduces a kink in the helix located approximately at the midpoint of the domain. The C-terminal domain was modelled using AlphaFold [98]. The calnexin C-terminal domain undergoes distinct post-translational modifications including palmitoylation at Cys^482^ and Cys^483^ [35] (shown in blue); sumoylation at Lys^505^ [96] (shown in green); and phosphorylation at Ser^534^, Ser^544^ and Ser^563^ [97] (shown in red); Asp^539^ proteolytic cleavage site (shown in black). Known and potential sites of post-translational modifications in the calnexin C-terminal domains of various species are shown in Supplementary Figure S1.

Meanwhile, the mouse calnexin gene (*Canx*) is located on a reverse strand of the long arm of murine chromosome 11 and has a similar arrangement but with only 14 exons that are transcribed into a 4281 bp transcript which encodes a polypeptide of 591 amino acid residues. The human calnexin polypeptide is a 67 kDa type-I integral membrane protein but is often mistakenly referred to as a 90 kDa protein due to its high content of acidic residues which electrostatically repel SDS resulting in an insufficient electromotive incentive and a lowered electrophoretic mobility on SDS-PAGE. The calnexin polypeptide is composed of three topological domains (Figure 2 and Figure 3): an N-terminal ER intraluminal domain, a transmembrane segment and a cytosol-facing C-terminal domain [16]. The ER luminal domain is responsible for chaperone function and thus is often referred to as the folding module [56,90]. The transmembrane segment anchors calnexin to the ER membrane and possibly also contributes to its chaperone function [91]. Finally, the 90 amino acid long C-terminal domain is oriented towards the cytosol and undergoes several distinct post-translational modifications [12,33,35,92,93,94,95,96,97].

### 3.1. The ER Lumen-Localized N-Terminal Domain

**Figure 4:**
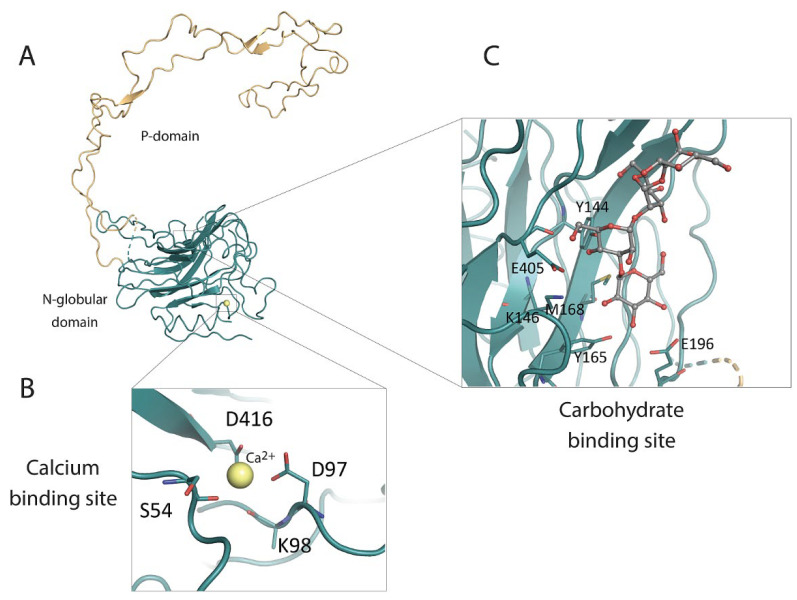
Crystal structure of the calnexin luminal domain and its characteristics. (**A**) Crystal structure of the calnexin intraluminal domain. The globular N-globular domain is shown in green while the P-domain is depicted in yellow. (**B**) Calnexin putative Ca^2+^-binding site, showing a Ca^2+^ ion (yellow circle) coordinated by Asp^416^, Asp^97^, Ser^54^ and potentially Lys^98^. (**C**) Calnexin carbohydrate binding site, showing the sidechains of Tyr^144^, Lys^146^, Tyr^165^, Glu^196^, Glu^405^ and Met^168^ involved in the binding of carbohydrate moieties.

The ER luminal domain of calnexin is responsible for its lectin-like chaperone function and is the site for interaction with cyclophilin B, ERp29 and ERp57 [56,57,58,59,60]. It also contains a 20 amino acid residue N-terminal signal sequence that is responsible for targeting calnexin into the ER. The crystal structure of the intraluminal portion of canine calnexin was solved by Schrag et al. [99] at 2.9 Å resolution and revealed the asymmetry featuring two distinct structural components comprised of a compact globular domain towards the N-terminus (referred to as the N-domain) and an elongated arm-like proline-rich domain (referred to as the P-domain) towards the C-terminus (Figure 4) [99]. The N-domain is composed of concave and convex antiparallel β sheets that have six and seven β strands, respectively, which together form a β-sandwich structure [99]. The calnexin luminal domain co-crystallized with α-D-glucose revealed the site for the carbohydrate binding within N-domain on the concave β sheet, where Tyr^144^, Lys^146^, Tyr^165^, Glu^196^ and Glu^405^ (coordinates refer to the human calnexin amino acid sequence) form hydrogen bonds with glucose hydroxyl groups, while the Met^168^ sidechain interacts with the glucose ring via van der Waals interactions [99].

The P-domain [100] extends 140 Å away from the N- domain and forms a large hairpin loop (Figure 4). This loop consists of two types of motifs (Motif 1 and Motif 2) of proline-rich sequence repeats bearing the consensus sequence of I-DP(D/E)A-KPEDWD(D/E) and G-W-P-IN-P-Y, respectively. Each motif is repeated four times and arranged in a linear manner ‘11112222’ where four repeats of motif 1 extend away from the N-globular domain and then fold back onto the strand with four repeats of motif 2. Every copy of motif 1 interacts with a copy of motif 2 in a head-to-tail fashion [99]. This hook-like arm is further stabilized via hydrophobic interactions of conserved isoleucine residues [26]. In addition, there are three regions of high amino acid sequence similarity, flanking the repeat motifs. Both N-globular and P-domain harbors one disulfide bond: Cys^140^–Cys^174^ and Cys^340^–Cys^346^, respectively, in the human calnexin sequence [99].

Early studies indicated that calnexin harbous multiple low affinity Ca^2+^ binding sites in both the N- and C-terminal regions [16,89]. However, the three-dimensional structure of the luminal domain revealed only a single putative Ca^2+^ binding site coordinated by Asp^416^, Asp^97^ and Ser^54^ [99]. This Ca^2+^ binding site is highly conserved between calnexin and calreticulin, as the P-domain of calreticulin also binds a single Ca^2+^ ion with high affinity [101] through Asp^328^ (refers to human calreticulin; homologous to Asp^416^ in calnexin), Gln^26^, Lys^62^, and Lys^64^, with two water molecules [99]. Moreover, the binding of Ca^2+^ to the ER luminal portion of calnexin plays a structural role by triggering Ca^2+^-dependent conformational changes [102]. In addition, the calnexin luminal domain was shown to bind Zn^2+^ and ATP; both regulate conformational changes [102], while ATP alone enhances the aggregation suppression abilities in vitro, even though no ATPase activity has been reported [103], and Zn^2+^ facilitates the binding of ERp57 [104].

### 3.2. The Transmembrane Domain

Using a molecular dynamics simulation, a single transmembrane spanning domain comprised on an α-helix was predicted for calnexin. The Pro^494^ (refers to the human calnexin sequence) at approximately the midpoint of the α-helix introduces a tilt of ~30° with respect to the surface of the membrane (Figure 3) [34]. Replacement of the Pro^494^ with leucine to remove the kink in the transmembrane helix negatively affects the interaction between calnexin and the ribosome–translocon complex [34]. It was proposed that the anchoring of calnexin to the ER membrane facilitates its association with membrane-bound substrates, enhancing its chaperone function [91].

### 3.3. The Cytosolic C-Terminal Domain

Much of the work characterizing the function of calnexin has focused on the intraluminal and transmembrane domain and consequently little is known about the C-terminal domain. It is known that the C-terminal domain plays an important role in the retention of calnexin in the ER as this domain contains the RKPRRE motif, which acts as an ER retention sequence [105]. To date, no structural information about the calnexin C-terminal domain is available. This highly acidic 90 amino acid long segment (theoretical pI of 4.52 for human calnexin) faces the cytosol and is thought to be flexible and unstructured [16,26]. The acidic nature of this domain contributes to the unusual electrophoretic mobility of the calnexin in SDS-PAGE. Based on the number of amino acids encoded by the calnexin mRNA, the predicted molecular mass of calnexin is 67 kDa. However, the apparent molecular mass of calnexin on SDS-PAGE gels is dramatically increased to 90 kDa protein. The anionic character of the calnexin C-terminal domain imparts this domain with multiple low affinity, but high capacity Ca^2+^-binding sites [87].

## 4. Post-Translational Modifications of the Calnexin C-Terminal Domain

Unlike the compact luminal domain of calnexin, the cytosol-exposed C-terminal domain appears to be unstructured which makes it easily accessible. Indeed, recent studies have found that the cytosol-exposed C-terminal domain undergoes post-translational modifications, which include palmitoylation [92,93,94], phosphorylation, sumoylation [12,33,35,95,96,97] and proteolytic cleavage [10] (Figure 3).

### 4.1. Palmitoylation

Calnexin is palmitoylated at both juxtamembranous cysteines Cys^502^ and Cys^503^ (or Cys^482^ and Cys^483^ counting from the mature N-terminus) by an ER palmitoyltransferase DHHC6 [34]. Over 90% of calnexin molecules are S-acylated at a steady state suggesting that the cell maintains constant pamitoylation-depalmitoylation cycles of calnexin [34]. Moreover, molecular dynamics simulations predicted that upon palmitoylation, the C-terminal domain adopts different conformations with respect to the transmembrane helix axis suggesting that palmitoylation might affect the capacity and/or selectivity of calnexin to interact with additional proteins outside the ER via its cytosolic C-terminal domain. Palmitoylation at Cys^503^ was predicted to have a more prominent effect on the conformation of the C-terminal domain, suggesting possible functional/regulatory difference between Cys^502^ and Cys^503^ state of palmitoylation. Using both computational and experimental approaches, it has been shown that the calnexin half-life increases 9-fold upon palmitoylation [106]. As a functional consequence, the palmitoylation of both cysteines preferentially localizes calnexin to the perinuclear rough ER while also facilitating the association with the ribosome–translocon complex. This association was shown to be crucial for chaperone function, as calnexin can capture its substrates as they emerge through translocon [34]. Other studies have also shown that palmitoylated calnexin is localized to the mitochondria-ER contact sites [107,108], where it interacts and controls sarco-endoplasmic reticulum Ca^2+^ transport ATPase 2b (SERCA2b) [13], an interaction that modulates ER-mitochondria Ca^2+^ signaling [11,12,35]. It has been shown that upon short-term ER stress, the pool of palmitoylated calnexin is reduced, shifting non-palmitoylated calnexin localization to rough ER where it interacts with ERp57 to facilitate protein folding and quality control [12]. Further studies should help to clarify the impact of calnexin redistribution on the remodeling of cellular processes and the regulation of cellular function.

### 4.2. Phosphorylation

Calnexin harbors three phosphorylation sites in its C-terminal domain: Ser^534^, Ser^544^ and Ser^563^ (in the human calnexin sequence). These sites are known to be phosphorylated by casein kinase CK2 (at Ser^534^ and Ser^544^) [97,109] and extracellular signal-regulated kinase-1 ERK-1 (at Ser^563^) [33]. ERK-1 is activated by mitogen-activated protein kinase 1 MEK1 under conditions that promote protein misfolding [110]; therefore, the phosphorylation state of Ser^563^ exemplifies calnexin function in ER quality control. Upon ER stress, detection of misfolded protein accumulation leads to ERK-1 activation and enhanced calnexin phosphorylation at Ser^563^ which in turn leads to the specific recruitment of calnexin to ER-membrane bound ribosomes. This specific recruitment facilitates calnexin function as a chaperone to enhance glycoprotein quality control at the ER ribosome–translocon complex by prolonging its association with unfolded proteins [33,95]. In synergy with the phosphorylation of Ser^563^, the phosphorylation of Ser^534^ and Ser^544^ by CK2 further promotes calnexin–ribosome interaction [33]. Moreover, the phosphorylation of these two residues disrupts calnexin interaction with phosphofurin acidic cluster sorting protein 2 (PACS-2), a key regulator of mitochondria/ER contact sites [111], thus shifting calnexin distribution from these sites to rough ER [39]. Another study also showed that calcineurin, a Ca^2+^-dependent phosphatase, dephosphorylates Ser^563^ [112] thereby controlling the phosphorylation status of Ser^563^ which was shown to also modulate calnexin interaction with SERCA2b which in turn regulate intracellular Ca^2+^ oscillations [13]. Taken together, phosphorylation of calnexin in its C-terminal domain illustrates another level of complexity in controlling calnexin distribution among different ER membrane subdomains and therefore influences its function.

### 4.3. SUMOylation

Sumoylation is a type of post-translational modification involving the covalent attachment of the small ubiquitin-related modifier (SUMO) protein (∼10 kDa) to certain proteins [113]. Calnexin interacts with sumoylation E2 ligase UBC9 via its C-terminal domain and undergoes sumoylation at Lys^505^ [96]. Sumoylated calnexin interacts with protein tyrosine phosphatase 1B PTP1B [96], linking the protein quality control pathway with insulin and leptin signaling [114].

### 4.4. Proteolytic Cleavage

It was previously reported that apoptotic stimuli caused calnexin to undergo proteolytic cleavage at its C-terminal domain by either caspase-3 or caspase-4 at a DXXD site resulting in the attenuation of apoptosis [9]. Another study showed that cells stimulated with epidermal growth factor (EGF) triggers caspase-8-dependent proteolytic cleavage of the calnexin C-terminal domain at Asp^519^ yielding a 63 amino acid peptide [10] that translocates to the nucleus where it makes a stable complex with Protein Inhibitor of Activated STAT 3 (PIAS3) thus preventing PIAS3 from inhibiting Signal Transducer and Activator of Transcription 3 (STAT3). Palmitoylation of calnexin, which targets it to mitochondria/ER contact sites, is required for EGF-induced cleavage of calnexin whereas ER stress prevents its proteolytic cleavage [10].

 

Despite some variation in the calnexin C-terminal primary structure (Figure S1), the positions of Cys^502^, Cys^503^, Ser^563^, Ser^534^, Ser^544^, Lys^505^, which represent sites for post-translational modifications (Figure 3), are moderately conserved (ranging from 44 to 65%) suggesting similar modifications might also occur in different organisms.

## 5. The Calnexin C-Terminal Domain, a Cytosol-ER Regulatory Nexus

In addition to modifying enzymes, recent studies have found that the C-terminal domain of calnexin interacts with a variety of proteins [14,96,115,116,117,118] which, depending on the type of modifications introduced, have a substantial impact on cellular homeostasis and function. Some of the cellular processes affected by the post-translational modification of the C-terminal domain have already been described in the preceding section.

Endocytosis is an example of a cellular process that is altered via the calnexin C-terminal domain. It has been found that the binding of SGIP1 to the calnexin C-terminal domain inhibits clathrin-dependent endocytosis in neuronal cells, and the absence of calnexin in mice causes an increased endocytosis in the nervous system [15]. Cellular efflux of substrate cholesterol is similarly altered via the calnexin C-terminal domain. The binding of the human immunodeficiency virus (HIV) protein Nef to ABCA1, the main cellular cholesterol transporter, disrupts the interaction of calnexin with ABCA1, leading to its retention in the ER and eventual degradation, thereby inhibiting ABCA1-dependent cholesterol efflux [119]. The accumulation of HIV-infected cells accumulate cholesterol in HIV infected cells leads to the increased formation of plasma membrane lipid rafts which serve as sites of HIV entry, assembly, and budding [120]. Moreover, Nef promotes the interaction calnexin with the HIV glycoprotein protein gp160 enhancing HIV envelope protein maturation [119].

Further insight into the pathophysiological relevance of the calnexin C-terminal domain was provided by recent studies on a complex formed by the calnexin C-terminal domain and the cytosolic protein Fabp5 (also referred to as epidermal fatty acid binding protein). This unexpected complex was detected during a search for calnexin interaction partners using the yeast two-hybrid system [118]. While whole-body calnexin deficiency leads to myelinopathy in mice [48], loss of calnexin also causes resistance to induction of experimental autoimmune encephalomyelitis (EAE), a model of inflammatory central nervous system demyelination, coincident with the phenotype of whole-body Fabp5 deficiency [121,122]. In fact, deletion of the calnexin cytosolic C-terminal domain, the site for Fabp5 interaction, is sufficient to impart resistance to EAE induction [51]. This resistance due to the inhibition of circulating T-cell infiltration across endothelial cells of the blood–brain barrier when the formation of the complex between calnexin C-terminal domain and Fabp5 was prevented [51,121]. In contrast, experiments using a cell culture model of the blood–brain barrier demonstrated that promoting the formation of the calnexin/Fabp5 complex enabled T-cells to traverse the endothelial cells of the blood–brain barrier model [51]. It is possible that the stable interaction of Fabp5 with the calnexin C-terminal domain prevents calnexin from interacting with other regulatory proteins or from redistributing to subdomains of the ER membrane system.

The ability of the calnexin C-terminal domain to undergo distinct post-translational modifications and interact with regulatory proteins suggests that the calnexin C-terminal domain is a dynamically modifiable segment of a key ER-resident protein that acts as an interface for facilitating communication between cytosolic and ER processes.

## 6. Summary

Calnexin was first characterized as a molecular chaperone in the ER. The structure of calnexin resembles calreticulin, another ER-resident chaperone and Ca^2+^-binding protein. Calnexin and calreticulin, along with additional accessory ER proteins that include ERp57, participate in the calnexin/calreticulin cycle responsible for the folding and maturation of glycosylated proteins synthesized in the ER some of which are destined for cellular export. Unlike calreticulin however, calnexin is anchored to the ER membrane via its transmembrane domain and its C-terminal domain that extends to the cytosol. Recent studies have discovered that the calnexin C-terminal domain is subject to post-translational modification featuring lipidation, sumoylation, phosphorylation and proteolytic cleavage. These modifications result in the redistribution of calnexin within ER membranes and are associated with the remodelling of cellular processes. Other cytosolic proteins have also been found to interact with the calnexin C-terminal domain and influence cellular function, indicating that these interactions are important in integrating cytosolic and ER events. These findings help pave the way towards the identification and characterization of new calnexin functions, in addition to its well-recognized role as a molecular chaperone.

## Data Availability

No new experimental data were created or analyzed in this study. Data sharing is not applicable to this article.

## Supplementary Materials

The following supporting information can be downloaded at: https://www.mdpi.com/article/10.3390/cells12030403/s1, Figure S1: Amino acid sequence alignment of calnexin from multiple species.

## References

[1] Fagone P., Jackowski S. (2009). Membrane phospholipid synthesis and endoplasmic reticulum function. J. Lipid Res..

[2] Braakman I., Hebert D.N. (2013). Protein folding in the endoplasmic reticulum. Cold Spring Harb. Perspect. Biol..

[3] Clapham D.E. (2007). Calcium signaling. Cell.

[4] Kulak N.A., Geyer P.E., Mann M. (2017). Loss-less Nano-fractionator for High Sensitivity, High Coverage Proteomics. Mol. Cell Proteom..

[5] Anelli T., Sitia R. (2008). Protein quality control in the early secretory pathway. EMBO J..

[6] Anfinsen C.B. (1973). Principles that govern the folding of protein chains. Science.

[7] Balchin D., Hayer-Hartl M., Hartl F.U. (2016). In vivo aspects of protein folding and quality control. Science.

[8] Ellgaard L., McCaul N., Chatsisvili A., Braakman I. (2016). Co- and Post-Translational Protein Folding in the ER. Traffic.

[9] Takizawa T., Tatematsu C., Watanabe K., Kato K., Nakanishi Y. (2004). Cleavage of calnexin caused by apoptotic stimuli: Implica-tion for the regulation of apoptosis. J. Biochem..

[10] Lakkaraju A.K., van der Goot F.G. (2013). Calnexin controls the STAT3-mediated transcriptional response to EGF. Mol. Cell.

[11] Gutiérrez T., Qi H., Yap M.C., Tahbaz N., Milburn L.A., Lucchinetti E., Lou P.H., Zaugg M., LaPointe P.G., Mercier P. (2020). The ER chaperone calnexin controls mitochondrial positioning and respiration. Sci. Signal..

[12] Lynes E.M., Raturi A., Shenkman M., Ortiz Sandoval C., Yap M.C., Wu J., Janowicz A., Myhill N., Benson M.D., Camp-bell R.E. (2013). Palmitoylation is the switch that assigns calnexin to quality control or ER Ca^2+^ signaling. J. Cell Sci..

[13] Roderick H.L., Lechleiter J.D., Camacho P. (2000). Cytosolic phosphorylation of calnexin controls intracellular Ca^2+^ oscillations via an interaction with SERCA2b. J. Cell Biol..

[14] Li H.D., Liu W.X., Michalak M. (2011). Enhanced clathrin-dependent endocytosis in the absence of calnexin. PLoS ONE.

[15] Louvard D., Reggio H., Warren G. (1982). Antibodies to the Golgi complex and the rough endoplasmic reticulum. J. Cell Biol..

[16] Wada I., Rindress D., Cameron P.H., Ou W.J., Doherty J.J., Louvard D., Bell A.W., Dignard D., Thomas D.Y., Ber-geron J.J. (1991). SSR alpha and associated calnexin are major calcium binding proteins of the endoplasmic reticulum membrane. J. Biol. Chem..

[17] Degen E., Williams D.B. (1991). Participation of a novel 88-kD protein in the biogenesis of murine class I histocompatibility mole-cules. J. Cell Biol..

[18] Hochstenbach F., David V., Watkins S., Brenner M.B. (1992). Endoplasmic reticulum resident protein of 90 kilodaltons associates with the T- and B-cell antigen receptors and major histocompatibility complex antigens during their assembly. Proc. Natl. Acad. Sci. USA.

[19] Michalak M., Groenendyk J., Szabo E., Gold L.I., Opas M. (2009). Calreticulin, a multi-process calcium-buffering chaperone of the endoplasmic reticulum. Biochem. J..

[20] Ahluwalia N., Bergeron J.J., Wada I., Degen E., Williams D.B. (1992). The p88 molecular chaperone is identical to the endoplasmic reticulum membrane protein, calnexin. J. Biol. Chem..

[21] David V., Hochstenbach F., Rajagopalan S., Brenner M.B. (1993). Interaction with newly synthesized and retained proteins in the endoplasmic reticulum suggests a chaperone function for human integral membrane protein IP90 (calnexin). J. Biol. Chem..

[22] Cala S.E., Ulbright C., Kelley J.S., Jones L.R. (1993). Purification of a 90-kDa protein (Band VII) from cardiac sarcoplasmic reticu-lum. Identification as calnexin and localization of casein kinase II phosphorylation sites. J. Biol. Chem..

[23] Coe H., Michalak M. (2010). ERp57, a multifunctional endoplasmic reticulum resident oxidoreductase. Int. J. Biochem. Cell Biol..

[24] Hammond C., Braakman I., Helenius A. (1994). Role of N-linked oligosaccharide recognition, glucose trimming, and calnexin in glycoprotein folding and quality control. Proc. Natl. Acad. Sci. USA.

[25] Helenius A. (1994). How N-linked oligosaccharides affect glycoprotein folding in the endoplasmic reticulum. Mol. Biol. Cell.

[26] Kozlov G., Gehring K. (2020). Calnexin cycle-structural features of the ER chaperone system. FEBS J..

[27] Parodi A.J. (2000). Protein glucosylation and its role in protein folding. Annu. Rev. Biochem..

[28] Caramelo J.J., Parodi A.J. (2008). Getting in and out from calnexin/calreticulin cycles. J. Biol. Chem..

[29] Helenius A., Aebi M. (2004). Roles of N-linked glycans in the endoplasmic reticulum. Annu. Rev. Biochem..

[30] Lamriben L., Graham J.B., Adams B.M., Hebert D.N. (2016). N-Glycan-based ER Molecular Chaperone and Protein Quality Con-trol System: The Calnexin Binding Cycle. Traffic.

[31] Hebert D.N., Molinari M. (2007). In and out of the ER: Protein folding, quality control, degradation, and related human diseases. Physiol. Rev..

[32] Agellon L.B., Michalak M. (2017). The Endoplasmic Reticulum and the Cellular Reticular Network. Adv. Exp. Med. Biol..

[33] Chevet E., Wong H.N., Gerber D., Cochet C., Fazel A., Cameron P.H., Gushue J.N., Thomas D.Y., Bergeron J.J. (1999). Phos-phorylation by CK2 and MAPK enhances calnexin association with ribosomes. EMBO J..

[34] Lakkaraju A.K., Abrami L., Lemmin T., Blaskovic S., Kunz B., Kihara A., Dal Peraro M., van der Goot F.G. (2012). Palmitoylated calnexin is a key component of the ribosome-translocon complex. EMBO J..

[35] Lynes E.M., Bui M., Yap M.C., Benson M.D., Schneider B., Ellgaard L., Berthiaume L.G., Simmen T. (2012). Palmitoylated TMX and calnexin target to the mitochondria-associated membrane. EMBO J..

[36] Wiest D.L., Burgess W.H., McKean D., Kearse K.P., Singer A. (1995). The molecular chaperone calnexin is expressed on the sur-face of immature thymocytes in association with clonotype-independent CD3 complexes. EMBO J..

[37] Wiest D.L., Bhandoola A., Punt J., Kreibich G., McKean D., Singer A. (1997). Incomplete endoplasmic reticulum (ER) retention in immature thymocytes as revealed by surface expression of "ER-resident" molecular chaperones. Proc. Natl. Acad. Sci. USA.

[38] Okazaki Y., Ohno H., Takase K., Ochiai T., Saito T. (2000). Cell surface expression of calnexin, a molecular chaperone in the endo-plasmic reticulum. J. Biol. Chem..

[39] Myhill N., Lynes E.M., Nanji J.A., Blagoveshchenskaya A.D., Fei H., Carmine Simmen K., Cooper T.J., Thomas G., Sim-men T. (2008). The subcellular distribution of calnexin is mediated by PACS-2. Mol. Biol. Cell.

[40] Chen Y., Ma D., Wang X., Fang J., Liu X., Song J., Li X., Ren X., Li Q., Li Q. (2019). Calnexin Impairs the Antitumor Im-munity of CD4+ and CD8+ T Cells. Cancer Immunol. Res..

[41] Kobayashi M., Nagashio R., Jiang S.X., Saito K., Tsuchiya B., Ryuge S., Katono K., Nakashima H., Fukuda E., Goshima N. (2015). Calnexin is a novel sero-diagnostic marker for lung cancer. Lung Cancer.

[42] Thompson J.D., Gibson T.J., Plewniak F., Jeanmougin F., Higgins D.G. (1997). The CLUSTAL_X windows interface: Flexible strat-egies for multiple sequence alignment aided by quality analysis tools. Nucleic Acids Res..

[43] Waterhouse A.M., Procter J.B., Martin D.M., Clamp M., Barton G.J. (2009). Jalview Version 2—A multiple sequence alignment edi-tor and analysis workbench. Bioinformatics.

[44] Rosenbaum E.E., Hardie R.C., Colley N.J. (2006). Calnexin is essential for rhodopsin maturation, Ca^2+^ regulation, and photorecep-tor cell survival. Neuron.

[45] Lee W., Lee T.H., Park B.J., Chang J.W., Yu J.R., Koo H.S., Park H., Yoo Y.J., Ahnn J. (2005). Caenorhabditis elegans calnexin is N-glycosylated and required for stress response. Biochem. Biophys. Res. Commun..

[46] Xu K., Tavernarakis N., Driscoll M. (2001). Necrotic cell death in C. elegans requires the function of calreticulin and regulators of Ca^2+^ release from the endoplasmic reticulum. Neuron.

[47] Hung I.C., Cherng B.W., Hsu W.M., Lee S.J. (2013). Calnexin is required for zebrafish posterior lateral line development. Int. J. Dev. Biol..

[48] Kraus A., Groenendyk J., Bedard K., Baldwin T.A., Krause K.-H., Dubois-Dauphin M., Dyck J., Rosenbaum E.E., Korn-gut L., Colley N.J. (2010). Calnexin deficiency leads to dysmyelination. J. Biol. Chem..

[49] Denzel A., Molinari M., Trigueros C., Martin J.E., Velmurgan S., Brown S., Stamp G., Owen M.J. (2002). Early postnatal death and motor disorders in mice congenitally deficient in calnexin expression. Mol. Cell Biol..

[50] Mesaeli N., Nakamura K., Zvaritch E., Dickie P., Dziak E., Krause K.H., Opas M., MacLennan D.H., Michalak M. (1999). Calre-ticulin is essential for cardiac development. J. Cell Biol..

[51] Paskevicius T., Jung J., Pujol M., Eggleton P., Qin W., Robinson A., Gutowski N., Holley J., Smallwood M., Newcombe J. (2020). The Fabp5/calnexin complex is a prerequisite for sensitization of mice to experimental autoimmune encephalomyeli-tis. FASEB J..

[52] Apweiler R., Hermjakob H., Sharon N. (1999). On the frequency of protein glycosylation, as deduced from analysis of the SWISS-PROT database. Biochim. Biophys. Acta.

[53] Kornfeld R., Kornfeld S. (1985). Assembly of asparagine-linked oligosaccharides. Annu. Rev. Biochem..

[54] Hebert D.N., Foellmer B., Helenius A. (1995). Glucose trimming and reglucosylation determine glycoprotein association with cal-nexin in the endoplasmic reticulum. Cell.

[55] Rodan A.R., Simons J.F., Trombetta E.S., Helenius A. (1996). N-linked oligosaccharides are necessary and sufficient for association of glycosylated forms of bovine RNase with calnexin and calreticulin. EMBO J..

[56] Zapun A., Darby N.J., Tessier D.C., Michalak M., Bergeron J.J., Thomas D.Y. (1998). Enhanced catalysis of ribonuclease B folding by the interaction of calnexin or calreticulin with ERp57. J. Biol. Chem..

[57] Frickel E.M., Riek R., Jelesarov I., Helenius A., Wuthrich K., Ellgaard L. (2002). TROSY-NMR reveals interaction between ERp57 and the tip of the calreticulin P-domain. Proc. Natl. Acad. Sci. USA.

[58] Kozlov G., Bastos-Aristizabal S., Määttänen P., Rosenauer A., Zheng F., Killikelly A., Trempe J.F., Thomas D.Y., Gehring K. (2010). Structural basis of cyclophilin B binding by the calnexin/calreticulin P-domain. J. Biol. Chem..

[59] Kozlov G., Muñoz-Escobar J., Castro K., Gehring K. (2017). Mapping the ER Interactome: The P Domains of Calnexin and Calre-ticulin as Plurivalent Adapters for Foldases and Chaperones. Structure.

[60] Sakono M., Seko A., Takeda Y., Ito Y. (2014). PDI family protein ERp29 forms 1:1 complex with lectin chaperone calreticulin. Biochem. Biophys. Res. Commun..

[61] Tannous A., Patel N., Tamura T., Hebert D.N. (2015). Reglucosylation by UDP-glucose:glycoprotein glucosyltransferase 1 delays glycoprotein secretion but not degradation. Mol. Biol. Cell.

[62] Plemper R.K., Wolf D.H. (1999). Retrograde protein translocation: ERADication of secretory proteins in health and disease. Trends Biochem. Sci..

[63] Cabral C.M., Choudhury P., Liu Y., Sifers R.N. (2000). Processing by endoplasmic reticulum mannosidases partitions a secretion-impaired glycoprotein into distinct disposal pathways. J. Biol. Chem..

[64] Hosokawa N., Wada I., Hasegawa K., Yorihuzi T., Tremblay L.O., Herscovics A., Nagata K. (2001). A novel ER alpha-mannosidase-like protein accelerates ER-associated degradation. EMBO Rep..

[65] Tremblay L.O., Herscovics A. (1999). Cloning and expression of a specific human alpha 1,2-mannosidase that trims Man_9_GlcNAc_2_ to Man_8_GlcNAc_2_ isomer B during N-glycan biosynthesis. Glycobiology.

[66] Meusser B., Hirsch C., Jarosch E., Sommer T. (2005). ERAD: The long road to destruction. Nat. Cell Biol..

[67] Jakob C.A., Burda P., Roth J., Aebi M. (1998). Degradation of misfolded endoplasmic reticulum glycoproteins in Saccharomyces cerevisiae is determined by a specific oligosaccharide structure. J. Cell Biol..

[68] Oda Y., Hosokawa N., Wada I., Nagata K. (2003). EDEM as an acceptor of terminally misfolded glycoproteins released from cal-nexin. Science.

[69] Molinari M., Calanca V., Galli C., Lucca P., Paganetti P. (2003). Role of EDEM in the release of misfolded glycoproteins from the calnexin cycle. Science.

[70] Christianson J.C., Ye Y. (2014). Cleaning up in the endoplasmic reticulum: Ubiquitin in charge. Nat. Struct. Mol. Biol..

[71] Yang M., Luo S., Wang X., Li C., Yang J., Zhu X., Xiao L., Sun L. (2021). ER-Phagy: A New Regulator of ER Homeostasis. Front. Cell Dev. Biol..

[72] Forrester A., De Leonibus C., Grumati P., Fasana E., Piemontese M., Staiano L., Fregno I., Raimondi A., Marazza A., Bruno G. (2019). A selective ER-phagy exerts procollagen quality control via a Calnexin-FAM134B complex. EMBO J..

[73] Fregno I., Fasana E., Bergmann T.J., Raimondi A., Loi M., Soldà T., Galli C., D’Antuono R., Morone D., Danieli A. (2018). ER-to-lysosome-associated degradation of proteasome-resistant ATZ polymers occurs via receptor-mediated vesicular transport. EMBO J..

[74] Diedrich G., Bangia N., Pan M., Cresswell P. (2001). A role for calnexin in the assembly of the MHC class I loading complex in the endoplasmic reticulum. J. Immunol..

[75] Jackson M.R., Cohen-Doyle M.F., Peterson P.A., Williams D.B. (1994). Regulation of MHC class I transport by the molecular chap-erone, calnexin (p88, IP90). Science.

[76] Suh W.K., Mitchell E.K., Yang Y., Peterson P.A., Waneck G.L., Williams D.B. (1996). MHC class I molecules form ternary com-plexes with calnexin and TAP and undergo peptide-regulated interaction with TAP via their extracellular domains. J. Exp. Med..

[77] Zhang Q., Tector M., Salter R.D. (1995). Calnexin recognizes carbohydrate and protein determinants of class I major histocompati-bility complex molecules. J. Biol. Chem..

[78] Vassilakos A., Cohen-Doyle M.F., Peterson P.A., Jackson M.R., Williams D.B. (1996). The molecular chaperone calnexin facilitates folding and assembly of class I histocompatibility molecules. EMBO J..

[79] Prasad S.A., Yewdell J.W., Porgador A., Sadasivan B., Cresswell P., Bennink J.R. (1998). Calnexin expression does not enhance the generation of MHC class I-peptide complexes. Eur. J. Immunol..

[80] Sadasivan B.K., Cariappa A., Waneck G.L., Cresswell P. (1995). Assembly, peptide loading, and transport of MHC class I mole-cules in a calnexin-negative cell line. Cold Spring Harb. Symp. Quant. Biol..

[81] Anderson K.S., Cresswell P. (1994). A role for calnexin (IP90) in the assembly of class II MHC molecules. EMBO J..

[82] Kearse K.P., Williams D.B., Singer A. (1994). Persistence of glucose residues on core oligosaccharides prevents association of TCR alpha and TCR beta proteins with calnexin and results specifically in accelerated degradation of nascent TCR alpha proteins within the endoplasmic reticulum. EMBO J..

[83] Van Leeuwen J.E., Kearse K.P. (1996). Calnexin associates exclusively with individual CD3 delta and T cell antigen receptor (TCR) alpha proteins containing incompletely trimmed glycans that are not assembled into multisubunit TCR complexes. J. Biol. Chem..

[84] Gardner T.G., Franklin R.A., Robinson P.J., Pederson N.E., Howe C., Kearse K.P. (2000). T cell receptor assembly and expression in the absence of calnexin. Arch. Biochem. Biophys..

[85] Van Leeuwen J.E., Kearse K.P. (1996). The related molecular chaperones calnexin and calreticulin differentially associate with nas-cent T cell antigen receptor proteins within the endoplasmic reticulum. J. Biol. Chem..

[86] Grupp S.A., Mitchell R.N., Schreiber K.L., McKean D.J., Abbas A.K. (1995). Molecular mechanisms that control expression of the B lymphocyte antigen receptor complex. J. Exp. Med..

[87] Wu Y., Pun C., Hozumi N. (1997). Roles of calnexin and Ig-alpha beta interactions with membrane Igs in the surface expression of the B cell antigen receptor of the IgM and IgD classes. J. Immunol..

[88] Foy S.P., Matsuuchi L. (2001). Association of B lymphocyte antigen receptor polypeptides with multiple chaperone proteins. Immunol. Lett..

[89] Tjoelker L.W., Seyfried C.E., Eddy R.L., Byers M.G., Shows T.B., Calderon J., Schreiber R.B., Gray P.W. (1994). Human, mouse, and rat calnexin cDNA cloning: Identification of potential calcium binding motifs and gene localization to human chromosome 5. Biochemistry.

[90] Oliver J.D., Roderick H.L., Llewellyn D.H., High S. (1999). ERp57 functions as a subunit of specific complexes formed with the ER lectins calreticulin and calnexin. Mol. Biol. Cell.

[91] Ho S.C., Rajagopalan S., Chaudhuri S., Shieh C.C., Brenner M.B., Pillai S. (1999). Membrane anchoring of calnexin facilitates its interaction with its targets. Mol. Immunol..

[92] Dowal L., Yang W., Freeman M.R., Steen H., Flaumenhaft R. (2011). Proteomic analysis of palmitoylated platelet proteins. Blood.

[93] Kang R., Wan J., Arstikaitis P., Takahashi H., Huang K., Bailey A.O., Thompson J.X., Roth A.F., Drisdel R.C., Mastro R. (2008). Neural palmitoyl-proteomics reveals dynamic synaptic palmitoylation. Nature.

[94] Yount J.S., Moltedo B., Yang Y.Y., Charron G., Moran T.M., López C.B., Hang H.C. (2010). Palmitoylome profiling reveals S-palmitoylation-dependent antiviral activity of IFITM3. Nat. Chem. Biol..

[95] Cameron P.H., Chevet E., Pluquet O., Thomas D.Y., Bergeron J.J. (2009). Calnexin phosphorylation attenuates the release of par-tially misfolded alpha1-antitrypsin to the secretory pathway. J. Biol. Chem..

[96] Lee D., Kraus A., Prins D., Groenendyk J., Aubry I., Liu W.-X., Li H.-D., Julien O., Touret N., Sykes B.D. (2015). UBC9-dependent association between calnexin and protein tyrosine phosphatase 1B (PTP1B) at the endoplasmic reticulum. J. Biol. Chem..

[97] Wong H.N., Ward M.A., Bell A.W., Chevet E., Bains S., Blackstock W.P., Solari R., Thomas D.Y., Bergeron J.J. (1998). Conserved in vivo phosphorylation of calnexin at casein kinase II sites as well as a protein kinase C/proline-directed kinase site. J. Biol. Chem..

[98] Jumper J., Evans R., Pritzel A., Green T., Figurnov M., Ronneberger O., Tunyasuvunakool K., Bates R., Zidek A., Potapenko A. (2021). Highly accurate protein structure prediction with AlphaFold. Nature.

[99] Schrag J.D., Bergeron J.J., Li Y., Borisova S., Hahn M., Thomas D.Y., Cygler M. (2001). The Structure of calnexin, an ER chaper-one involved in quality control of protein folding. Mol. Cell.

[100] Trombetta E.S., Helenius A. (1998). Lectins as chaperones in glycoprotein folding. Curr. Opin. Struct. Biol..

[101] Baksh S., Michalak M. (1991). Expression of calreticulin in Escherichia coli and identification of its Ca^2+^ binding domains. J. Biol. Chem..

[102] Ou W.J., Bergeron J.J., Li Y., Kang C.Y., Thomas D.Y. (1995). Conformational changes induced in the endoplasmic reticulum lu-minal domain of calnexin by Mg-ATP and Ca^2+^. J. Biol. Chem..

[103] Ihara Y., Cohen-Doyle M.F., Saito Y., Williams D.B. (1999). Calnexin discriminates between protein conformational states and functions as a molecular chaperone in vitro. Mol. Cell.

[104] Leach M.R., Cohen-Doyle M.F., Thomas D.Y., Williams D.B. (2002). Localization of the lectin, ERp57 binding, and polypeptide binding sites of calnexin and calreticulin. J. Biol. Chem..

[105] Rajagopalan S., Xu Y., Brenner M.B. (1994). Retention of unassembled components of integral membrane proteins by calnexin. Science.

[106] Dallavilla T., Abrami L., Sandoz P.A., Savoglidis G., Hatzimanikatis V., van der Goot F.G. (2016). Model-Driven Understanding of Palmitoylation Dynamics: Regulated Acylation of the Endoplasmic Reticulum Chaperone Calnexin. PLoS Comput. Biol..

[107] Hayashi T., Su T.P. (2007). Sigma-1 receptor chaperones at the ER-mitochondrion interface regulate Ca^2+^ signaling and cell survival. Cell.

[108] De Brito O.M., Scorrano L. (2010). An intimate liaison: Spatial organization of the endoplasmic reticulum-mitochondria relation-ship. EMBO J..

[109] Ou W.J., Thomas D.Y., Bell A.W., Bergeron J.J. (1992). Casein kinase II phosphorylation of signal sequence receptor alpha and the associated membrane chaperone calnexin. J. Biol. Chem..

[110] Nguyên D.T., Kebache S., Fazel A., Wong H.N., Jenna S., Emadali A., Lee E.H., Bergeron J.J., Kaufman R.J., Larose L. (2004). Nck-dependent activation of extracellular signal-regulated kinase-1 and regulation of cell survival during endoplasmic reticulum stress. Mol. Biol. Cell.

[111] Li C., Li L., Yang M., Zeng L., Sun L. (2020). PACS-2: A key regulator of mitochondria-associated membranes (MAMs). Pharmacol. Res..

[112] Bollo M., Paredes R.M., Holstein D., Zheleznova N., Camacho P., Lechleiter J.D. (2010). Calcineurin interacts with PERK and dephosphorylates calnexin to relieve ER stress in mammals and frogs. PLoS ONE.

[113] Geiss-Friedlander R., Melchior F. (2007). Concepts in sumoylation: A decade on. Nat. Rev. Mol. Cell Biol..

[114] Feldhammer M., Uetani N., Miranda-Saavedra D., Tremblay M.L. (2013). PTP1B: A simple enzyme for a complex world. Crit. Rev. Biochem. Mol. Biol..

[115] Dudek E., Millott R., Liu W.X., Beauchamp E., Berthiaume L.G., Michalak M. (2015). N-Myristoyltransferase 1 interacts with cal-nexin at the endoplasmic reticulum. Biochem. Biophys. Res. Commun..

[116] Hunegnaw R., Vassylyeva M., Dubrovsky L., Pushkarsky T., Sviridov D., Anashkina A.A., Üren A., Brichacek B., Vassylyev D.G., Adzhubei A.A. (2016). Interaction Between HIV-1 Nef and Calnexin: From Modeling to Small Molecule In-hibitors Reversing HIV-Induced Lipid Accumulation. Arterioscler. Thromb. Vasc. Biol..

[117] Myrum C., Soulé J., Bittins M., Cavagnini K., Goff K., Ziemek S.K., Eriksen M.S., Patil S., Szum A., Nair R.R. (2017). Arc Interacts with the Integral Endoplasmic Reticulum Protein, Calnexin. Front. Cell Neurosci..

[118] Jung J., Wang J., Groenendyk J., Lee D., Michalak M., Agellon L.B. (2017). Fatty acid binding protein (Fabp) 5 interacts with the calnexin cytoplasmic domain at the endoplasmic reticulum. Biochem. Biophys. Res. Commun..

[119] Jennelle L., Hunegnaw R., Dubrovsky L., Pushkarsky T., Fitzgerald M.L., Sviridov D., Popratiloff A., Brichacek B., Buk-rinsky M. (2014). HIV-1 protein Nef inhibits activity of ATP-binding cassette transporter A1 by targeting endoplasmic reticulum chaperone calnexin. J. Biol. Chem..

[120] Waheed A.A., Freed E.O. (2009). Lipids and membrane microdomains in HIV-1 replication. Virus Res..

[121] Jung J., Eggleton P., Robinson A., Wang J., Gutowski N., Holley J., Newcombe J., Dudek E., Paul A.M., Zochodne D. (2018). Calnexin is necessary for T cell transmigration into the central nervous system. JCI Insight.

[122] Rao E., Singh P., Li Y., Zhang Y., Chi Y.I., Suttles J., Li B. (2015). Targeting epidermal fatty acid binding protein for treatment of experimental autoimmune encephalomyelitis. BMC Immunol..

