# Supplementary material for: Calnexin, More Than Just a Molecular Chaperone
Source: Cells. 2023 Jan 24;12(3):403. doi: 10.3390/cells12030403 (PMC9913998; doi:10.3390/cells12030403)
Supplement: Supplementary file 1 [file Suppl_Figure_S1.pdf › Suppl_Figure_S1.pdf]

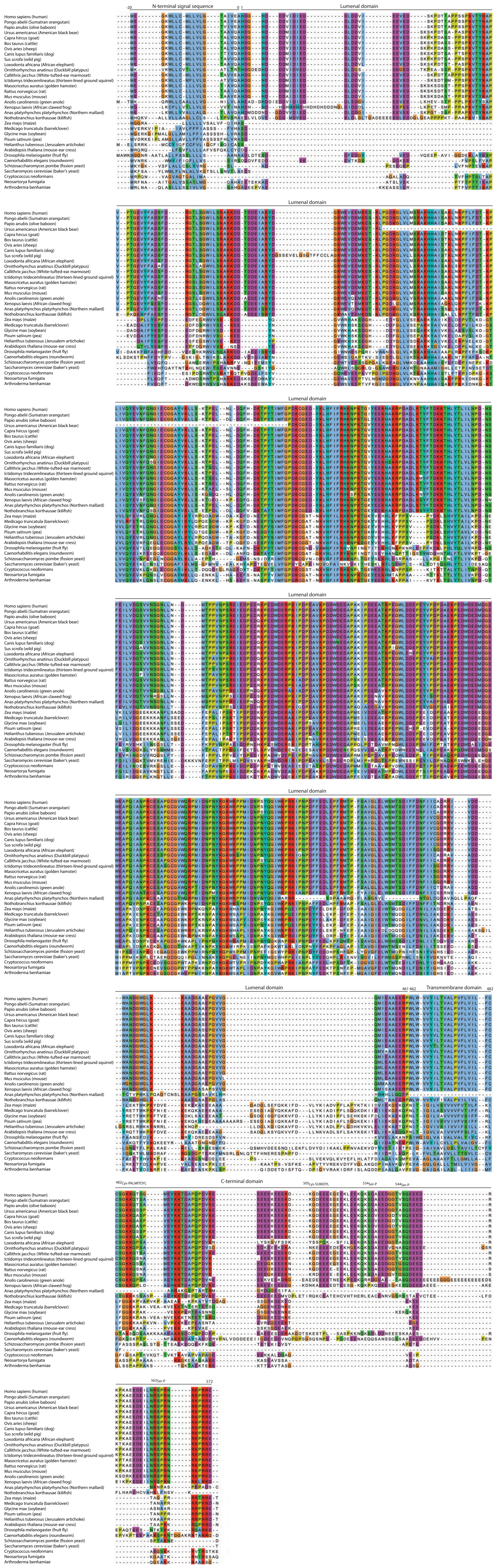
N-terminal signal sequence
Lumenal domain
Transmembrane domain
C-terminal domain
-20
0 1
461 462
482
482Cys-PALMITOYL
505Lys-SUMOYL
534Ser-P
544Ser-P
563Ser-P
572
Homo sapiens (human)
Pongo abelii (Sumatran orangutan)
Papio anubis (olive baboon)
Ursus americanus (American black bear)
Capra hircus (goat)
Bos taurus (cattle)
Ovis aries (sheep)
Canis lupus familiaris (dog)
Sus scrofa (wild pig)
Loxodonta africana (African elephant)
Ornithorhynchus anatinus (Duckbill platypus)
Callithrix jacchus (White-tufted-ear marmoset)
Ictidomys tridecemlineatus (thirteen-lined ground squirrel)
Masocricetus auratus (golden hamster)
Rattus norvegicus (rat)
Mus musculus (mouse)
Anolis carolinensis (green anole)
Xenopus laevis (African clawed frog)
Anas platyrhynchos platyrhynchos (Northern mallard)
Nothobranchius korthausae (kilifish)
Zea mays (maize)
Medicago truncatula (barrelclover)
Glycine max (soybean)
Pisum sativum (pea)
Helianthus tuberosus (Jerusalem artichoke)
Arabidopsis thaliana (mouse-ear cress)
Drosophila melanogaster (fruit fly)
Caenorhabditis elegans (roundworm)
Schizosaccharomyces pombe (fission yeast)
Saccharomyces cerevisiae (baker's yeast)
Cryptococcus neoformans
Neosartorya fumigata
Arthroderma benhamiae
